# Micro Vacuum Chuck and Tensile Test System for Bio-Mechanical Evaluation of 3D Tissue Constructed of Human Induced Pluripotent Stem Cell-Derived Cardiomyocytes (hiPS-CM)

**DOI:** 10.3390/mi10070487

**Published:** 2019-07-19

**Authors:** Kaoru Uesugi, Fumiaki Shima, Ken Fukumoto, Ayami Hiura, Yoshinari Tsukamoto, Shigeru Miyagawa, Yoshiki Sawa, Takami Akagi, Mitsuru Akashi, Keisuke Morishima

**Affiliations:** 1 Department of Mechanical Engineering, Osaka University, 2-1 Yamada-oka, Suita, Osaka 565-0871, Japan; 2 Global Center for Medical Engineering and Informatics, Osaka University, 2-1 Yamada-oka Suita, Osaka 565-0871, Japan; 3 Building Block Science Joint Research Chair, Graduate School of Frontier Biosciences, Osaka University, 1-3 Yamada-oka, Suita, Osaka 565-0871, Japan; 4 Department of Cardiovascular Surgery, Graduate School of Medicine, Osaka University, 2-2 Yamada-oka, Suita, Osaka 565-0871, Japan

**Keywords:** beating force, bio-mechanical property, cardiac 3D tissue, human induced pluripotent Stem cell-derived cardiomyocytes (hiPS-CM), tissue engineering, vacuum chuck

## Abstract

In this report, we propose a micro vacuum chuck (MVC) which can connect three-dimensional (3D) tissues to a tensile test system by vacuum pressure. Because the MVC fixes the 3D tissue by vacuum pressure generated on multiple vacuum holes, it is expected that the MVC can fix 3D tissue to the system easily and mitigate the damage which can happen by handling during fixing. In order to decide optimum conditions for the size of the vacuum holes and the vacuum pressure, various sized vacuum holes and vacuum pressures were applied to a normal human cardiac fibroblast 3D tissue. From the results, we confirmed that a square shape with 100 µm sides was better for fixing the 3D tissue. Then we mounted our developed MVCs on a specially developed tensile test system and measured the bio-mechanical property (beating force) of cardiac 3D tissue which was constructed of human induced pluripotent stem cell-derived cardiomyocytes (hiPS-CM); the 3D tissue had been assembled by the layer-by-layer (LbL) method. We measured the beating force of the cardiac 3D tissue and confirmed the measured force followed the Frank-Starling relationship. This indicates that the beating property of cardiac 3D tissue obtained by the LbL method was close to that of native cardiac tissue.

## 1. Introduction

Three-dimensional (3D) tissues which are constructed by cells in the environment *in vitro* have been applied in a wide range of fields such as regenerative medicine [1,2,3,4,5,6,7,8,9,10,11,12,13,14,15,16,17,18,19,20,21,22,23,24,25,26], drug development [2,9,11,12,13,14,15,26,27,28,29,30,31,32,33,34,35,36,37,38,39,40,41,42], disease modelling for pathology [11,12,13,14,15,36,43,44,45,46], bioactuators [47,48,49,50,51,52], food industry [42,52,53,54,55,56], and BioArt [52,55,56,57,58,59,60,61,62]. With the advancement of 3D tissue technologies, evaluation methods for them have been demanded. Conventionally, analytical approaches such as biochemical, immunological, morphological [24,63], electrophysiological, and motion image analysis [31] methods have been applied to evaluate artificial tissues. These methods, however, cannot evaluate bio-mechanical properties directly.

To construct the 3D tissues, it is necessary to make not only biochemical and electrophysiological evaluations, but also bio-mechanical evaluations. By evaluating bio-mechanical properties of 3D tissues, various conditions of 3D tissues can be understood. For example, in the field of regenerative medicine, bio-mechanical evaluations are important for constructing 3D tissues which replicate living tissues. The behaviors of cells in tissues are affected by the surrounding environment [64,65]. If the surrounding environment affects the bio-mechanical properties in a different manner from that *in vivo*, tissues might develop in an undesirable form and might act in ways which are different from their true functions. The fact that conditions of the extracellular matrix influence bio-mechanical properties of 3D tissues also indicates the importance of measurement of 3D tissues [4,5,18,23]. When constructing thick tissues, a luminal structure is needed for nutritional transport [29]; hence, the luminal structure is an important parameter for 3D tissue construction. The bio-mechanical properties of 3D tissues are affected by the luminal structure because the mechanical properties of the luminal structure and other components of the tissues are each different. Thus, measurement of bio-mechanical properties can be evaluated by using the luminal structure of 3D tissues. Additionally, it is important for transplantation of 3D tissues *in vitro* to ascertain whether or not the 3D tissues are broken by the *in vivo* pressure, whether or not the 3D tissues satisfy the desired mechanical properties, and whether or not the 3D tissues have the optimum stiffness or viscoelasticity. For the above reasons, various researchers have statically measured bio-mechanical properties of artificial tissues such as bone [16,17], cartilage [18,19], tendon [20,21,22], vein [23], and skin [24,25]. Additionally, if large-scale manufacturing of 3D tissues is to be done, automatic handling of 3D tissues may be demanded, and then, information on mechanical properties may be important also. In the field of drug development, bio-mechanical properties are also important for the reasons described above. In the food industry field, artificial meats must possess mechanical properties (texture) similar to that of real meats [54,55]. In the field of BioArt, to get the sense of touch for semi-living, that is, artificial, tissues, it is necessary to start creating some sort of bond [56]. Because the sense of touch mainly depends on mechanical information, the mechanical properties of artificial 3D tissues must be known.

As mentioned above, the bio-mechanical properties are important parameters for constructing 3D tissues. However, conventional methods, such as biochemical and electrophysiological methods and microscopic observations have not been able to evaluate bio-mechanical properties of 3D tissues. Therefore, we tried to measure bio-mechanical properties of a sheet shape tissue (a cell sheet). For example, we measured the adhesion force of a cell sheet by the ninety-degree peel test with our newly developed system [66,67,68]. We also measured stiffness of a cell sheet with a tensile test system which we developed [69,70]. Since the procedures for the ninety-degree peel test and tensile test are defined by the International Standard Organization (ISO), a proposed method using them should be standardized easily.

Next, we looked at the importance of mechanical properties of cardiac 3D tissue beating. The bio-mechanical properties such as adhesion force and stiffness, which we measured previously, were static properties. Nowadays, dynamical bio-mechanical property, such as beating force, is beginning to receive attention with the appearance of cardiac 3D tissues which have been realized by tissue engineering technologies, and embryo-stem (ES) cell and induced pluripotent stem cell (iPSC) technologies. For example, cardiac 3D tissues were constructed by a layer-by-layer (LbL) method [26,31]. Thus, various methods which can evaluate beating properties of cardiac 3D tissues which were constructed by various methods have been proposed and beating force of cardiac 3D tissues has been measured [3,4,5,6,7,8,9,10,11,12,13,15,32,33,34,35,36,37,43,44,45,46]. The cardiac 3D tissues assembled by the LbL method were evaluated by biochemical, immunological, electrophysiological, and motion image analysis methods [31]. However, these evaluation methods for cardiac 3D tissues which were constructed by LbL method cannot evaluate bio-mechanical properties directly. Therefore, our objective in this report was to overcome this deficiency.

However, fixing 3D tissues onto a measurement system is difficult because of their mechanical and chemical fragility. Many studies have tried to fix 3D tissues by various methods [3,4,5,6,7,8,9,10,11,12,13,15,32,33,34,35,36,37,43,44,45,46]. These methods have limits to the size and the shape of the 3D tissues because of how the fixing to the force measurement system is done. Therefore, in the measurement of mechanical properties for cardiac 3D tissues, most cardiac 3D tissues for which mechanical properties were measured did not consist of just cells. These 3D tissues consisted of cells and scaffold (e.g., fibrin gel and collagen gel). And even though cardiac 3D tissues consisted mainly of cells, the sizes of those 3D tissues were small (length, smaller than 2 mm; width, 200 µm) [37]. Additionally, these fixing methods needed a lot of preparation steps and complex operations.

Evaluation for dynamical bio-mechanical properties of cardiac 3D tissues which consist of just cells (for example, tissue obtained by the LbL method) and whose size is large has been needed. In order to mount the 3D tissue on the evaluation system, a fixture which enables fixing of various sizes and various shaped 3D tissues is necessary. Additionally, for reliable and efficient measurement, the fixture demands easy connection of the 3D tissues to the evaluation system. Therefore, the objective of this study was development of a system which can solve these issues.

In this study, we newly developed a special chucking tool and a tensile test system. The chucking tool can chuck the 3D tissue by vacuum pressure which is loaded through multi micro meter size holes. The tensile system can measure a small force (from sub-micro newton to milli newton level) and can drive the vacuum chucking tool. To confirm applicability of the system, we measured beating property of cardiac 3D tissue that consisted mostly of cells (with a tiny amount of extracellular matrix) and the tissue size was larger than that of conventional studies (diameter: 12 mm).

## 2. Materials and Methods 

### 2.1. Design of the Micro Vacuum Chuck (MVC)

In order to apply a tensile test for 3D tissues, tissues have to be connected to the tensile test system by using a chucking tool. In general, the mechanical and chemical strengths of conventionally chucked samples (i.e., metal, latex, plastic, cloth, and ceramic, etc.) are larger than those of 3D tissues and the sample sizes (millimeters to centimeters) are also bigger than those of 3D tissues (micrometers to millimeters). Thus, when conventional samples are connected to the tensile test system, a clamp type fixture or chemical bonding is applied. On the other hand, because 3D tissues are very soft (mechanically fragile), the clamp can crush them and chemical bonding can cause chemical damage to them. Additionally, the small 3D tissue size makes a fixing operation which employs the above methods difficult. Some reports which measured contractile force of 3D tissues have used fixing by piercing or hooking with a fine hook [3,4,5,6,7,8,12,37], tying with nylon fiber [10,13,46], embedding a special fixture in an early preparation stage [11,15,32,35,43,44,45], and using a silicon post (a micro pillar) [9,33,34,36] in a manner similar to the embedding method. We have also employed hooking for fixing sheet shape tissues [66,67,68,69,70]. However, when applying these fixing methods to the tensile test of 3D tissue, there are some problems. For instance, by hooking or piercing the 3D tissue, it may tear away from the pierced holes. Hooking also restricts the shape of the 3D tissues somewhat; however, a ring and a fiber (rod) shape are possible examples. When using the ring shape 3D tissue, mechanical properties due to the shape and friction between the hook and tissue become a concern. Tying is a complicated operation and it does not allow evaluation of many 3D tissues. Additionally, tying causes variation of the measured force and load mechanical damage to 3D tissues. Operations such as hooking, piercing, and tying may also cause unsure measurements because of deformation of the 3D tissue by stress concentration. To use a special embedded fixture, the shape of the 3D tissue and preparation method are restricted. Force generated by the 3D tissue cannot be measured directly by the micro pillar. It is difficult to apply the tensile test for the micro pillar since the length of the 3D tissues cannot change due to the structure of the system. Additionally, for the micro pillar, shapes of 3D tissues are limited (i.e., a fiber (rod) shape).

**Figure 1:**
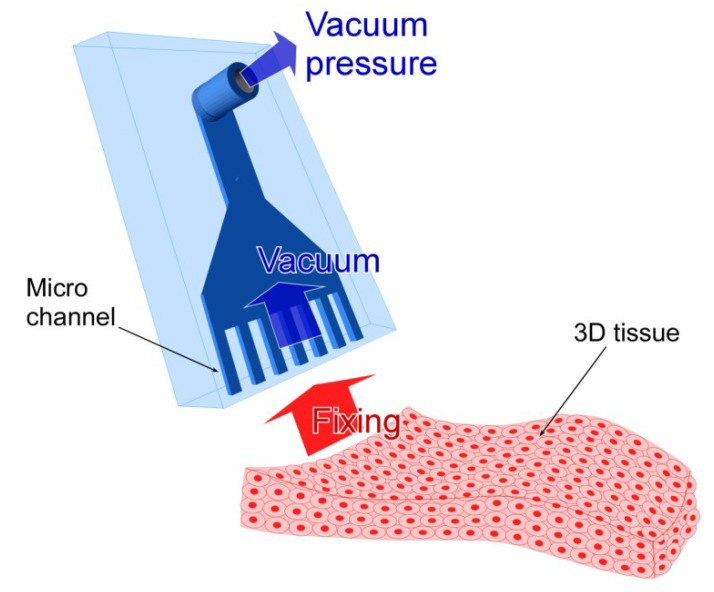
Conceptual illustration of the micro vacuum chuck (MVC). The 3D tissue is suctioned toward the micro size vacuum holes and fixed there by vacuum pressure which is generated at the holes.

Therefore, in this report, we have suggested an MVC which can fix 3D tissue by using vacuum pressure (Figure 1). The MVC has four advantages. First, the MVC can fix various shaped 3D tissues such as sheet, tube, and block shapes. Second, the MVC can fix 3D tissues easily without complex operations. Third, the MVC causes less damage to the 3D tissues and the uncertainty of the measurement is less by decreasing the effect of stress concentration because the MVC does not use fixation tools such as the fine hook, tying fiber, and embedding fixture. Finally, the MVC causes less chemical damage to the 3D tissue since the MVC does not need chemical bonding.

The 3D tissue was fixed with a negative pressure generated at vacuum holes of the MVC. When the single vacuum hole was applied for tissue fixing, an excessive vacuum was confirmed to cause a load on the tissue [71]. The excessive vacuum could cause damage to the 3D tissue, including unexpected length changes and deformation. Additionally, when fixing the 3D tissue with the single vacuum hole, the 3D tissue has to be detached from the culture surface before fixing. Therefore, the initial length of the 3D tissue changes by pre-detachment of the 3D tissue because the 3D tissue length is not fixed by the fixtures in advance. If the 3D tissue is fixed by using the single vacuum hole, the 3D tissue becomes detached from the vacuum hole when the 3D tissue is detached from the culture surface because the vacuum pressure which is generated by only one hole is weak (that is, the vacuum pressure is not strong enough to pull the 3D tissue into the vacuum hole). In order to fix the 3D tissue to the tensile test system while maintaining the initial length of the 3D tissue, a fixture which has multi vacuum holes is needed.

Therefore, we used the MVC with several vacuum holes. By increasing the number of vacuum holes, the 3D tissue could be fixed with a small stress concentration. Additionally, we expect that the excessive vacuum on the 3D tissue would be decreased because multiple close-by areas of the tissue would be pulled into the micro vacuum holes at the same time. By increasing the number of the vacuum holes, we can also expect certainty of fixation. Additionally, unlike hooking and tying, the MVC is able to fix variously sized and shaped 3D tissues by adjusting size and shape of the MVC. While, the method which uses an embedding special fixture may fix variously sized and shaped 3D tissues, it has to use impurities (e.g., fibrin gel and collagen gel) for formation of 3D tissues. 

**Figure 2:**
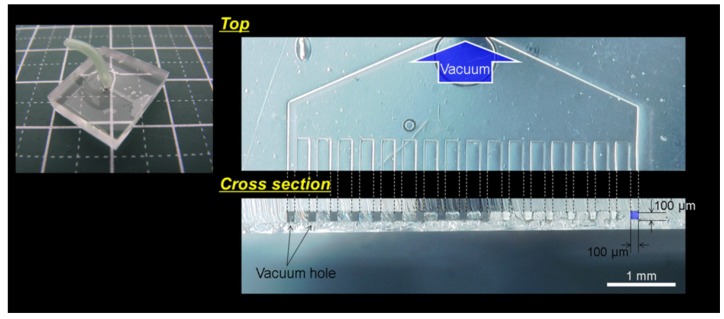
Photo of a micro vacuum check (MVC). The MVC had 17 micro vacuum holes and each hole was a square shape with a side length of 100 μm. The MVC was 5 mm wide.

Figure 2 shows a photo of the MVC. The MVC had 17 vacuum holes. The distance between the vacuum holes was 206 µm. Each vacuum hole was square and each side length was 100 μm. The vacuum hole size was decided by an experiment (see Section 2.3, Section 3.1, and Section 4). The MVC width was 5 mm. Because the single hole type vacuum chucks were made of fine tube, fabrication of the multi hole type vacuum chuck was difficult. We used a micro fluid channel fabricated by a photolithography technique (see Supplementary Materials and Figure S1). 

### 2.2. Design of Tensile Test System

**Figure 3:**
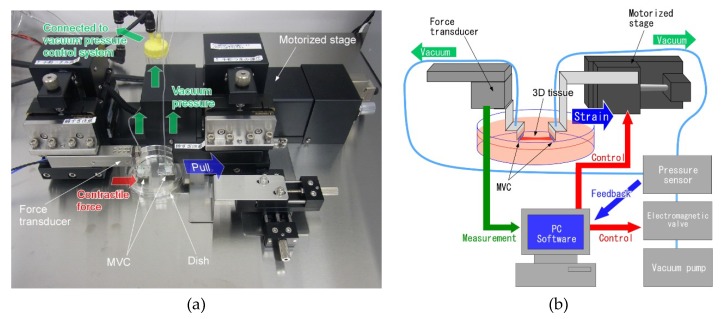

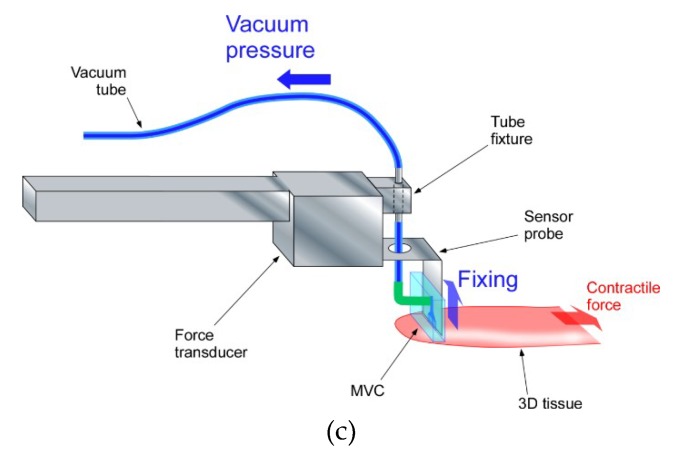
(**a**) Photo of the newly developed tensile test system with attached micro vacuum chuck (MVCs). (**b**) The tensile test system for 3D tissues consisted of six parts. (1) The force transducer was used to measure the bio-mechanical properties of the 3D tissue. (2) The MVCs fixed the 3D tissue to the force transducer and motorized stage. (3) The vacuum pressure generating system adjusted the vacuum pressure of the MVCs. This vacuum pressure system had a vacuum pump, an electromagnetic valve and a pressure sensor. (4) The motorized stage loaded tensile force onto the 3D tissue. (5) The culture dish protected the 3D tissue from drying out during the tensile test. (6) The charge-coupled device (CCD) camera and microscope were used in recording the test conditions. (**c**) Schematic illustration around one MVC connected to the force transducer of the tensile test system. The vacuum tube (a flexible silicon tube) was positioned vertically relative to the tensile force loaded direction. A tube fixture kept the tube in place mechanically.

Since there were no tensile test systems for 3D tissue, we also developed a special tensile test system. We designed the system configuration and devices conditions and ordered the system fabrication from a maker (HS101, Tech Alpha, Tokyo, Japan) (Figure 3a). The tensile test system for 3D tissues consisted of the six parts indicated in Figure 3b. (1) The force transducer was used to measure contractile force of the 3D tissue. (2) The MVCs fixed the 3D tissue to the force transducer and motorized stage. (3) The vacuum pressure generating system adjusted the vacuum pressure of the MVCs. This vacuum pressure system was constructed with a vacuum pump, an electromagnetic valve and a pressure sensor. The vacuum pressure was feedback controlled. (4) The motorized stage loaded a tensile force onto the 3D tissue. (5) The culture dish protected the 3D tissue from drying out during the tensile test. (6) The charge-coupled device (CCD) camera and microscope were used in recording the test conditions. Measured force data were analyzed by commercial analysis software (Igor Pro, Wave Metrics, Lake Oswego, OR, USA). Video data and measurement data were synchronized by turning on an LED lamp and recording the LED voltage signal simultaneously.

Figure 3c shows a schematic illustration of the region around one MVC which was connected to the force transducer of the tensile test system. In order to get close contact between the micro vacuum holes and the 3D tissue which adheres on the culture surface, the MVC was connected to the tensile test system vertically. When measuring the tensile force of the 3D tissue, stiffness of the vacuum tube which was supplying the vacuum pump suction at the MVC may affect the force measurement. Thus, a small diameter (ID, 0.5 mm; OD, 1 mm) and flexible silicon tube (CP-N-0.5-1-10, Shin-Etsu Polymer Co., Ltd., Tokyo, Japan) was used and the tube was positioned vertically relative to the tensile force loaded direction. If the tube connects the force transducer and the vacuum pressure generating system directly, the force transducer can measure mechanical noise (i.e., vibration) which is generated by the vacuum pump. Thus, a tube fixture kept the tube in place mechanically near the force transducer, and the tube fixture mechanically separated the force transducer from the vacuum pressure generating system.

### 2.3. Confirmation Experiment on the Effect of Vacuum Hole Size and Vacuum Pressure

To decide the optimum size of the vacuum hole of the MVCs, we confirmed amounts of 3D tissues which were pulled into variously sized vacuum holes at different vacuum pressures. We applied 3D tissues with pressures of 1 kPa, 3 kPa, 10 kPa, and 20 kPa. The square-shaped vacuum holes had side lengths of 50 µm, 100 µm and 200 µm. To simply evaluate these experimental results, we made MVCs with a single hole; the fabrication process was as described in the Supplementary Materials. 

The reason for setting the end of the vacuum pressure range as 20 kPa was as follows. In this study, we measured beating properties of cardiac 3D tissues with a loaded strain stimulation by tensile force. When a large strain stimulation is loaded, the 3D tissues become detached from the MVCs by the large tensile force and beating properties cannot be measured. Therefore, vacuum hole size and vacuum pressure needed to be determined in order to prevent the detachment of the 3D tissues from the MVCs by large strain stimulation. The normal human cardiac fibroblast (NHCF, CC-2509, Lonza, Basel, Switzerland) 3D tissue had a contractile force of about 400 µN (sample width, 3 mm; strain, 0.6) which was measured by using a prototype MVC and a prototype tensile test system [72]. If the contractile force increases linearly with increase of loaded strain, the contractile force could be about 667 µN when NHCF 3D tissue has a loaded strain of 1. In measurement of cardiac tissue contractile force for this study, we used 12-well cell culture inserts (diameter, 12 mm; this diameter would mean our tissue sample width was four times that of the reported sample width with presumed contractile force of about 667 µN). Thus, we hypothesized that the contractile force of cardiac tissue was about four times larger (about 2668 µN) than that of NHCF tissue for the loaded strain of 1 (667 µN). Since the number of vacuum holes of the multi-hole MVCs was 17 and the necessary fixing force was at least over 2668 µN, we hypothesized that the needed force generated by one vacuum hole was about 157 µN. Then, because the area of one vacuum hole was 0.01 µm^2^, the needed vacuum pressure was more than 15.7 kPa. Thus, we applied the maximum pressure of 20 kPa to the single-hole MVC. In this confirmation experiment, 3D tissues constructed from NHCF was used (see Supplementary Materials). 

The confirmation experiment was done in the culture medium (Dulbecco’s Modified Eagle Medium (D-MEM, High glucose, Nacalai Tesque, Kyoto, Japan) containing 10% fetal bovine serum (FBS, Life Technologies Co., Grand Island, NY, USA) at about 25 °C. About one minute after vacuuming NHCF 3D tissues using the single-hole MVCs, the amounts of pulled-in 3D tissue were observed through a microscope (Leica DMi1, Leica Microsystems, Tokyo, Japan) and recorded by the attached CCD camera. Then, recorded images were analyzed by the image processing software (ImageJ, 1.48v). After every observation was finished, the NHCF 3D tissue was removed from the single-hole MVCs, and then, another vacuuming and observation was made for the rest of the same 3D tissue. The amount of NHCF 3D tissue which was pulled into the single-hole MVC was defined as the distance between the tip of the pulled-in 3D tissue and the vacuum hole opening. Experiments were carried out once at each pressure and each size vacuum hole.

### 2.4. Tensile Test of 3D Tissue Constructed of Human Induced Pluripotent Stem Cell-Derived Cardiomyocytes (hiPS-CM)

**Figure 4:**
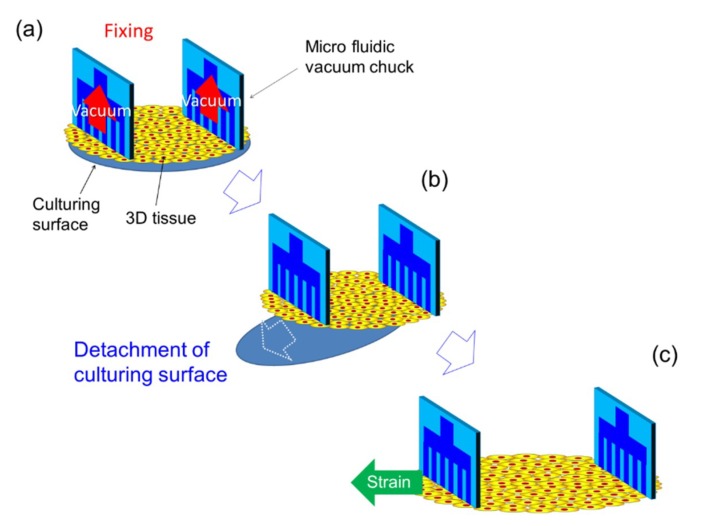
Schematic illustration of 3D tissue fixation by the micro vacuum chuck (MVC). (**a**) The 3D tissue, on a culturing surface that consisted of a culturing insert membrane, was fixed by the MVCs using the vacuum pressure. (**b**) The culturing surface was detached from the 3D tissue. (**c**) The 3D tissue was strained in one direction and beating force was measured.

In this test, 3D tissues constructed of human induced pluripotent stem cell-derived cardiomyocytes (hiPS-CM) were used (see Supplementary Materials). When cardiac 3D tissues are detached from the culturing surface, the 3D tissues shrink and their length changes. So first, the MVCs were used to hold the 3D tissue which was on the culturing surface that consisted of a culturing insert membrane by vacuum pressure (about 30 kPa) (Figure 4a). After confirming that the MVCs were holding the tissue, the culturing surface was detached from the adhered cardiac 3D tissue with a knife and tweezers (Figure 4b). By following these steps, we were able to measure the beating properties of the 3D tissue without any change in its conditions from those at the time of culturing. For example, the initial length of the 3D tissue was not changed because the length of the 3D tissue was fixed by the fixtures in advance. After again confirming the 3D tissue was held by the MVCs, the tissue was strained in one direction at a constant speed of 0.01 mm/s for arbitrary distances (Figure 4c). Then, after finishing the strain, the beating force was measured. The operations to strain the 3D tissue and measure the beating forces were repeated for each strain stimulation. Beating forces were measured for each strain stimulation without detachment of the 3D tissue. The extension was considered to be ended when the 3D tissue became detached from the MVCs. This detachment was judged from a rapid decrease of beating force of the 3D tissue or a decrease of vacuum pressure. The tensile test was done in the culture medium (DMEM + 10% FBS) at about 25 °C. In this experiment, the beating of 3D tissues was not evoked by such stimulations as electrical or optical stimulation. The passive contractile forces were measured. The initial length of the 3D tissue (distance between the MVCs) was 5 mm. Maximum strain value was 0.9. The force was measured at various strains before reaching the maximum strain. The beating force was defined as the value at the peak amplitude. The beating forces were measured from 8 to 20 times for each strain stimulation. We adjusted the number of measurements according to beat frequency. When the beating was slow, in order to shorten the experiment time, the number of beats for measurement was decreased. The measured peak forces were averaged and standard errors were also calculated. Three samples were used. Beating stress was calculated by force derived by the cross-sectional area of the 3D tissue. The slimmest parts of each 3D tissue were used for the cross-sectional area calculation. We assumed 89 µm as the thickness of all 3D tissues. The beating force measurement of the cardiac 3D tissues was conducted from 10 to 15 days after the seeding of cells in the culture inserts. The calculated beating stresses were averaged and standard errors were also calculated.

## 3. Results

### 3.1. Results of Confirmation Experiment on Effects of Vacuum Hole Size and Vacuum Pressure

**Figure 5:**
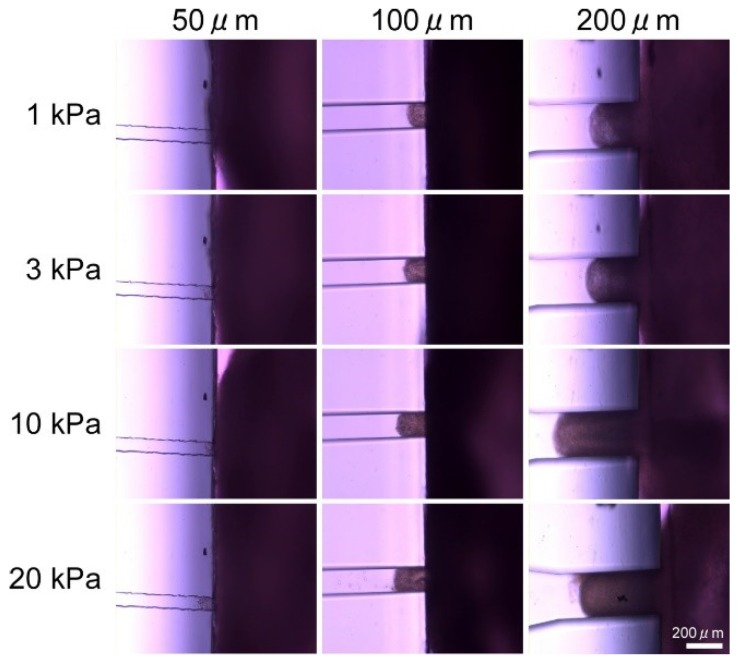
Photos of the normal human cardiac fibroblast (NHCF) 3D tissue that was pulled into the single-hole micro vacuum chuck (MVC). The cross-sectional shape of the vacuum hole was square and the side lengths were 50 µm, 100 µm and 200 µm. Applied vacuum pressures were 1 kPa, 3 kPa, 10 kPa, and 20 kPa. In each photo, a part of the NHCF 3D tissue was pulled into the hole. (Scale bar: 200 μm).

**Figure 6:**
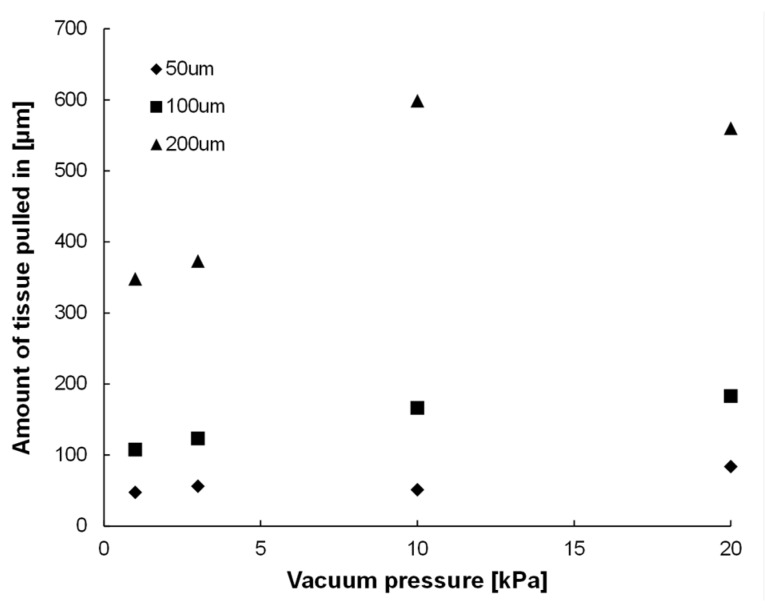
The amount of normal human cardiac fibroblast (NHCF) 3D tissues pulled into one vacuum hole by various vacuum pressures for three sizes of the square-shaped holes, 50 µm, 100 µm, and 200 µm. The amount was defined as the distance between the tip of the pulled-in NHCF 3D tissue and the hole opening. The amount of pulled-in 3D tissues tended to increase with increasing vacuum pressure, and it also increased with increasing size of the hole.

Figure 5 shows photos of a 3D tissue consisting of NHCF that was pulled into a single-hole MVC. As soon as the NHCF 3D tissue was attached on the MVC, part of it was pulled into the vacuum hole. Figure 6 shows the amount of NHCF 3D tissues which were pulled into the vacuum hole by various vacuum pressures for three sizes (side lengths) of the square-shaped holes, 50 µm, 100 µm, and 200 µm. The amount was defined as the distance between the tip of the pulled-in NHCF 3D tissue and the hole opening. We confirmed that the amount of pulled-in NHCF 3D tissues tended to increase with increment of vacuum pressure. Additionally, the amount of pulled-in NHCF 3D tissues also increased with increment of the vacuum hole size. When the hole was 50 µm × 50 µm, we observed collapse of the NHCF 3D tissue surface. 

### 3.2. Results of Tensile Test of 3D Tissue Constructed of Human Induced Pluripotent Stem Cell-Derived Cardiomyocytes (hiPS-CM)

**Figure 7:**
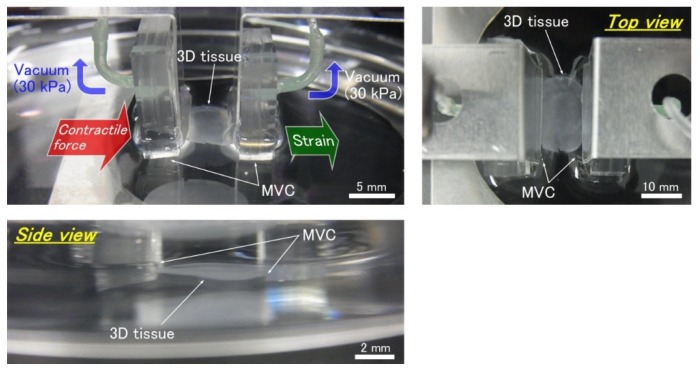
Photos of the cardiac 3D tissue fixed by the micro vacuum chuck (MVCs). Both ends of the cardiac 3D tissue were connected to the force transducer and the motorized stage with the MVCs which applied a vacuum pressure of 30 kPa.

**Figure 8:**
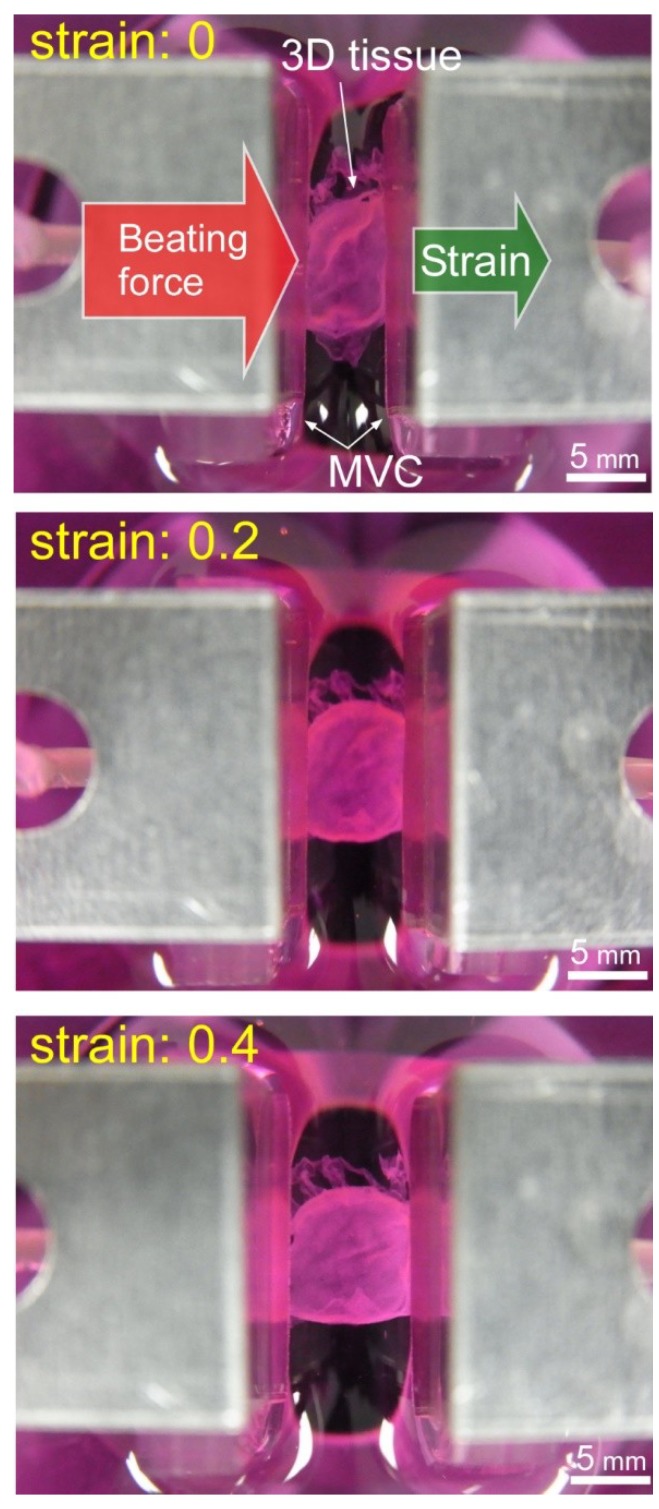
Photos of the cardiac 3D tissue during the tensile test.

**Figure 9:**
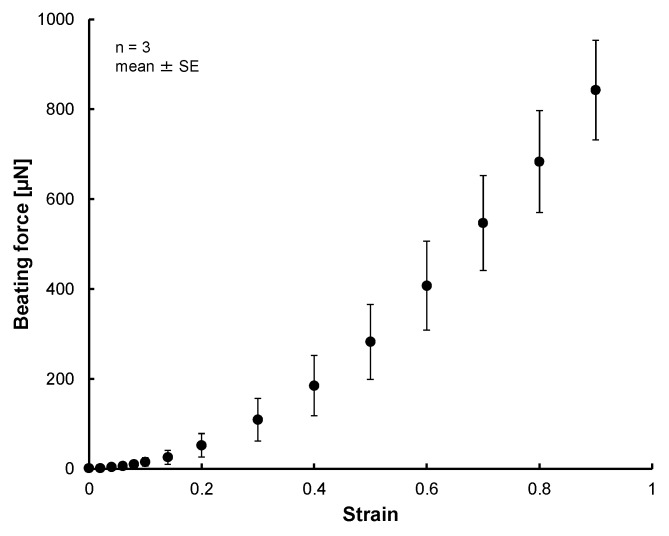
Strain-beating force properties of the cardiac 3D tissues. The beating forces were increased with the increment of strain.

**Figure 10:**
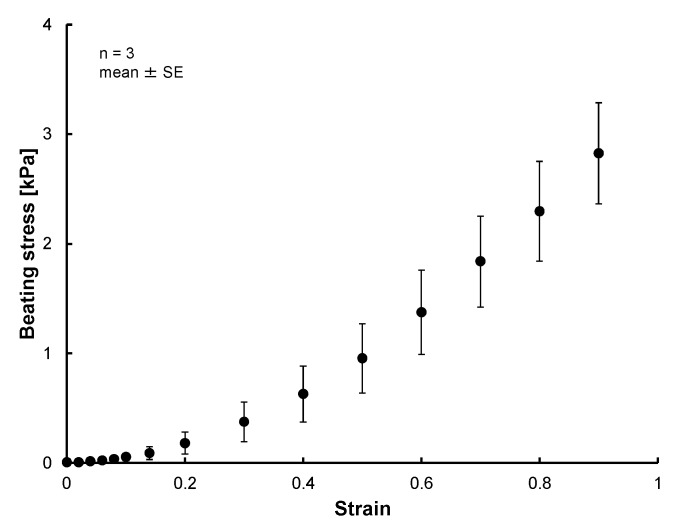
Strain-beating stress properties of the cardiac 3D tissues. The beating stress was increased with the increment of strain and the maximum beating stress was 2.8 ± 0.5 kPa (strain: 0.9).

The photo of Figure 7 shows the MVCs could fix the 3D tissue constructed of hiPS-CM to the force measurement system successfully. Figure 8 shows photos taken during the tensile test. The cardiac 3D tissue was fixed by the MVCs. Figure 9 shows strain-beating force properties of the cardiac 3D tissue. The maximum beating force of the 3D tissue was 843 ± 111 µN (strain: 0.9). All beating forces increased with increasing strain. When making a statistical analysis of beating force, we used the smallest value of the maximum strain among three samples for the common maximum value of strain. Figure 10 shows strain-beating stress properties of the cardiac 3D tissue. The average maximum stress was 2.8 ± 0.5 kPa (strain: 0.9).

## 4. Discussion

In evaluating 3D tissues, bio-mechanical properties are important. Therefore, we have applied the tensile test to the cardiac 3D tissue and measured beating force. In order to apply the tensile test to the 3D tissue, the tissue has to be connected to the tensile test system. However, there had been no fixture which was able to connect the 3D tissue to the tensile test system until our proposed system that held the tissue using vacuum pressure applied by an MVC. The MVC offers some advantages (see Section 2.1). In brief, these are the capabilities to: fix tissues in place without a complex operation; be applicable to variously sized and shaped 3D tissues; to mitigate damage caused to 3D tissue and uncertainty of the measurement; and to eliminate the chance of chemical damage.

We considered the detailed design of the MVC in an experiment which applied various vacuum hole sizes and various vacuum pressures to 3D tissues. In order to measure the beating properties of cardiac 3D tissues, a 3D tissue needs to be fixed using the MVCs even when tensile force is loaded to the 3D tissue. The MVCs must have sufficient vacuum pressure that can fix the 3D tissues. Because applying an excessive vacuum can cause damage to the tissue and unexpected length changes, a small amount of 3D tissue should be pulled into the vacuum hole by using the optimum hole size and shape for the MVC. By applying various sizes of vacuum holes, we observed that the amount of pulled-in NHCF 3D tissues increased with increment of vacuum hole size (Figure 5 and Figure 6). This indicated that the smaller size vacuum hole was better for fixing 3D tissues. The excessive vacuum condition for the smallest size MVC hole (50 µm) was smaller than that of other hole sizes. However, collapse of the NHCF 3D tissue surface was observed for this smallest size. The amount of excessive vacuum for the 200 µm hole MVC (max: 599) was 3.3 times larger than that of the 100 µm hole MVC (max: 183). Additionally, collapse of the NHCF 3D tissue surface was not observed for the 100 µm hole MVC. Thus, we concluded the MVC with the square hole size of 100 µm was the best for fixing 3D tissue.

Our developed MVC enables users to measure beating force of cardiac 3D tissue which is large sized and assembled from just cells by the LbL method. We observed that the cardiac 3D tissue was fixed by the MVC (Figure 7 and Figure 8). By preparing multiple vacuum holes, the 3D tissue was not pulled into the holes excessively compared to the single-hole MVC (Uesugi et al. [71] described the single-hole MVC). In the tensile test, the applied vacuum pressure was 30 kPa. This value was larger than the maximum vacuum pressure (20 kPa) which was applied in the confirmation experiment on the effect of vacuum hole size (see Section 3.1). We consider the reason why vacuum pressure was larger than 20 kPa. When the cardiac 3D tissue had a loaded tensile strain, a gap occurred between the 3D tissue and the holes of the MVC due to the contractile force and beating force of the extended 3D tissue, and then, vacuum pressure was leaked. This meant that the fixing ability of the MVC was decreased. Thus, we had to increase the applied vacuum pressure to compensate for the leakage effect of vacuum pressure. We considered the high vacuum pressure of 30 kPa was not a problem because the amount of pulled-in NHCF 3D tissues became fairly steady above 10 kPa, from which we hypothesized that the amount of pulled-in cardiac 3D tissue at 30 kPa was close to that of 10 kPa and 20 kPa.

The beating forces of cardiac 3D tissue samples increased with increase of strain (Figure 9). The trend we saw has also been seen for other artificial cardiac 3D tissues which consisted of iPSC-derived cells [8,10,12,15,37,46] and native cardiac tissues and cardiomyocytes [73,74,75,76]. This behavior is described by the Frank–Starling relationship [73,74,75,76]. This could indicate the possibility for the beating properties of LbL constricted tissue to be close to those of other artificial tissues and native tissues. There has been no study which confirmed the relationship of 3D cardiac tissues whose size was large and which consisted of just cells assembled by the LbL method. By using the MVC, we could measure the contractile properties of the 3D tissues whose size was large and which consisted of just cells. Since our system could measure the cardiac 3D tissue beating properties which were similar to those of native heart tissue, we anticipate that our system will also be able to evaluate the native-like drug response of cardiac 3D tissues in drug screening.

The beating stress of cardiac 3D tissues was 2.8 ± 0.5 kPa (strain: 0.9) (Figure 10). Conventional studies also have reported beating stress of iPS-derived cardiac tissues. The beating stress of cardiac 3D tissues consisting mainly of iPSC-derived cells (µHM) was about 4 mN/mm^2^ (beating of the tissues was controlled by applying a 1 Hz electrical stimulation without treatment of isoproterenol) [37]. The order of this stress value is close to our measured value. Because the beating force of µHM was increased with strain stimulation, the beating stress also increased further. The beating stresses of cardiac 3D tissues and cell sheet consisting of iPSC-derived cells and special gel have been reported as 0.08 [8], 0.62 [10], 1.34 [12], 3.3 [15] and 23.2 [11] mN/mm^2^. Compared with these values, our measured beating stress was an intermediate value. The values of these conventional studies varied. We consider that the reason for varying beating stresses was the various experimental conditions such as shape of the 3D tissues, culturing time, number of cells, cell species content, amount of ECM, ECM species content, extent of maturation, and fixing method. The cardiac 3D tissues which were cultured in the conventional studies might be loaded with a static contractile stimulation by self-contraction (self-organization) during culturing. Therefore, we cannot discuss in greater detail the beating stresses which were reported by conventional studies of artificial cardiac 3D tissues.

The beating stresses of human myocardium and myocyte have been reported as 44.0 mN/mm^2^ [77] and 51 mN/mm^2^ [78]. These amounts of beating stresses of native tissue and cells are over 10 times larger than ours. We consider that these differences were due to the extent of maturation, orientation, cell density, and composition of the ECM of cardiomyocytes.

## 5. Conclusions

In order to evaluate the bio-mechanical properties of a 3D tissue, the tensile test is applied. When applying the tensile test, the 3D tissues have to be fixed to a tensile test system by a special fixture. However, there had been no fixture which was able to fix variously sized and shaped 3D tissues at a small stress concentration easily. Therefore, we developed a micro vacuum chuck (MVC) which could fix the 3D tissue by vacuum pressure and we also developed a special tensile test system for it. In order to optimize the size of the vacuum holes and vacuum pressure, we applied various sized vacuum holes and vacuum pressures to a NHCF 3D tissue. The results showed that a square hole shape with 100 µm sides was better for fixing the 3D tissue. Then, we carried out the tensile test with cardiac 3D tissue which was assembled from hiPS-CMs by the layer-by-layer (LbL) method. We confirmed that the MVC could fix the cardiac 3D tissue during the tensile test. By using the newly developed tensile test system, the beating stress of cardiac 3D tissues was measured (2.8 ± 0.5 kPa, strain: 0.9). Additionally, the tensile test results for the cardiac 3D tissue confirmed that it followed the Frank–Starling relationship, and that cardiac 3D tissue assembled by the LbL method was close to native cardiac tissue.

Based on these results, we consider this bio-mechanical evaluation method which uses the MVC and the developed tensile test system is useful for evaluation of 3D tissues, and it can contribute to the fields of regenerative medicine, drug development, pathology, bioactuator development, food industry, and BioArt.

## Supplementary Materials

The following are available online at https://www.mdpi.com/2072-666X/10/7/487/s1, Figure S1: Schematic illustration showing assembly of the MVC.
