# Supplementary material for: Micro Vacuum Chuck and Tensile Test System for Bio-Mechanical Evaluation of 3D Tissue Constructed of Human Induced Pluripotent Stem Cell-Derived Cardiomyocytes (hiPS-CM)
Source: Micromachines (Basel). 2019 Jul 19;10(7):487. doi: 10.3390/mi10070487 (PMC6680730; doi:10.3390/mi10070487)
Supplement: Supplementary file 1 [file micromachines-10-00487-s001.pdf]

Supplementary Materials: Micro Vacuum Chuck and Tensile Test System for Bio-mechanical Evaluation of 3D Tissue Constructed of Human Induced Pluripotent Stem Cell-derived Cardiomyocytes (hiPS-CM)

Kaoru Uesugi, Fumiaki Shima, Ken Fukumoto, Ayami Hiura, Yoshinari Tsukamoto, Shigeru Miyagawa, Yoshiki Sawa, Takami Akagi, Mitsuru Akashi and Keisuke Morishima

Fabrication Method of the MVC

The fabrication process is described below. First, photoresist (SU-8 3050, Nippon Kayaku Co., Ltd., Tokyo, Japan) was spin coated (thickness: 100 μm) on a silicon wafer (Figure S1a). Then, the photoresist layer was exposed to ultraviolet light through a photomask (Figure S1b). The exposed photoresist was developed and a mold was formed (Figure S1c). Biocompatible silicon rubber, poly(dimethylsiloxane) (PDMS) (SILPOT 184, Dow Corning Toray Co., Ltd., Tokyo, Japan), was cast on the mold and baked at 120 °C (Figure S1d). After hardening of PDMS, the PDMS structure was removed from the mold (Figure S1e). Then, the PDMS structure and cover glass (C022241, Matsunami Glass ind., Ltd., Osaka, Japan) were both irradiated with an excimer light (SUS713, Ushio Inc., Tokyo, Japan) under a stereomicroscope that bonded them together (Figure S1f). In order to get complete bonding, the assembled MVC was baked at 110 °C for more than 30 min while pressing it with a force of several newtons.

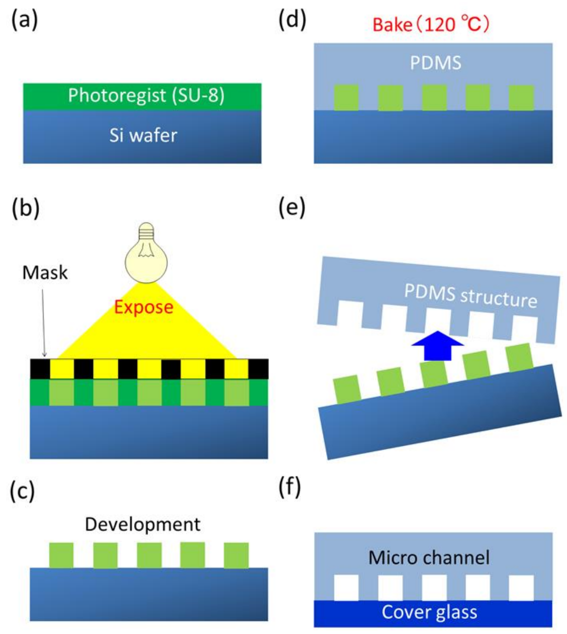

Figure 1. Schematic illustration showing assembly of the MVC. (**a**) Photoresist was spin coated (thickness: 100 μm) on silicon wafer. (**b**) Then, the photoresist layer was exposed to ultraviolet light through a photomask. (**c**) Exposed photoresist was developed and a mold was formed. (**d**) PDMS was cast on the mold and baked at 120 °C. (**e**) The PDMS structure was removed from the mold. (**f**) The PDMS structure and cover glass were both irradiated with excimer light under a stereomicroscope that bonded them together. In order to get complete bonding, the assembled MVC was baked at 110 °C for more than 30 min while pressing it.

Preparation of NHCF 3D Tissue Sample

They were constructed as described elsewhere [1]. Briefly, NHCF were coated with fibronectin (FN) and gelatin (G) alternately. Then, FN-G coated NHCF (1×10^6 cells) were cultured on 24-well cell culture inserts (culture area: 0.33 cm^2) with 0.4 μm pore size membrane (Transwell 3470, Corning Inc., Kennebunk, ME, USA) in 5 % CO_2 at 37 °C for several days. After 1 day of incubation, the NHCF multilayered tissues were assembled on the culturing insert membrane. For the confirmation experiment on the optimum size of vacuum holes, tissue samples were cultured for 5 days.

Preparation of Cardiac 3D Tissue Samples

The 3D cardiac tissue samples were constructed as described elsewhere [1]. In brief, FN-G coated human induced pluripotent stem cell-derived cardiomyocytes (hiPS-CM) [2], FN-G coated NHCF and human cardiac microvascular endothelial cells (HMVEC) were co-cultured on 12-well cell culture inserts (diameter, 12 mm; culture area, 1.12 cm^2) in 5 % CO_2 at 37 °C for 10 to 15 days. The numbers of cells contained in a well were 2.55×10^6 (hiPS-CM), 8.5×10^5 (NHCF) and 3.4×10^5 (HMVEC). After culturing cells, tissue samples equivalent to 10 layers were structured.

Reference

1. Amano, Y.; Nishiguchi, A.; Matsusaki, M.; Iseoka, H.; Miyagawa, S.; Sawa, Y.; Seo, M.; Yamaguchi, T.; Akashi, M. Development of vascularized iPSC derived 3D-cardiomyocyte tissues by filtration Layer-by-Layer technique and their application for pharmaceutical assays. *Acta Biomater.* **2016**, *33*, 110–121.
2. Sasano, Y.; Fukumoto, K.; Akagi, T.; Akashi, M. Convenient differentiation method for human cardiomyocytes from human induced pluripotent stem cells without embryoid body formation. In preparation.
